# Assessing the diagnostic accuracy of symptoms and signs of degenerative cervical myelopathy: A prospective study

**DOI:** 10.1038/s41598-025-20928-4

**Published:** 2025-10-23

**Authors:** Khadija Soufi, Omar Ortuno, Jose A. Castillo, Nádia F. Simões de Souza, Tess Perez, Giselle Ghabussi, Tiffany Chu, Kee D. Kim, Richard Price, Yashar Javidan, Hai V. Le, Rolando F. Roberto, Safdar Khan, Eric O. Klineberg, Lindsay Tetreault, Benjamin Davies, Carl M. Zipser, Aria Nouri, Shekar Kurpad, Bizhan Aarabi, Brian K. Kwon, Sukhvinder Kalsi-Ryan, Michael G. Fehlings, Allan R. Martin

**Affiliations:** 1 Spinal Cord Injury, Function, and Imaging (SCIFI) Laboratory, Department of Neurological Surgery, University of California, Davis, US; 2 Department of Neurosurgery, University Medical Center Groningen, University of Groningen, Groningen, Netherlands; 3 Creighton University School of Medicine, Omaha, Nebraska US; 4 Department of Orthopedic Surgery, University of California, Davis, Sacramento, California US; 5 Department of Orthopedic Surgery, McGovern School of Medicine, Houston, Texas US; 6 Department of Neurology, Massachusetts General Hospital, Boston, Massachusetts US; 7 Department of Neurology, Brigham & Women’s Hospital, Boston, Massachusetts US; 8 Department of Neurosurgery, Cambridge University Hospitals, Cambridge, England; 9 Department of Neurology, Balgrist University Hospital, Zurich, Switzerland; 10 Departement of Neurosurgery, University Hospital of Geneva, Geneva, Switzerland; 11 Department of Neurosurgery, Medical College of Wisconsin, Milwaukee, Wisconsin US; 12 Department of Neurosurgery, University of Maryland, College Park, Maryland, US; 13 Department of Neurosurgery, University of British Columbia, Vancouver, British Columbia US; 14 Spinal Cord Injury Center and Department of Neurology and Neurophysiology, Balgrist University Hospital, University of Zurich, Zurich, Switzerland; 15 KITE Research Institute, Toronto, Ontario Canada; 16 Department of Physical Therapy, UHN2, University of Toronto, Toronto, Ontario Canada; 17 Department of Surgery, University of Toronto, Toronto, Ontario Canada

**Keywords:** Degenerative cervical myelopathy, Diagnosis, Neurodegeneration, Spinal cord diseases

## Abstract

Degenerative cervical myelopathy (DCM) is a clinical diagnosis based on history, physical exam, and imaging, but standardized criteria have not been established, contributing to diagnostic delays. We conducted a prospective study of DCM and healthy subjects that comprehensively evaluated symptoms, patient-reported and clinician-administered outcome measures, and physical assessments of motor and sensory function.Diagnostic utility was evaluated using Youden’s Index (YI=sensitivity+specificity-1). 139 DCM patients and 108 age-matched healthy subjects were compared. Distinguishing symptoms included neck pain (YI=63%), upper extremity (UE) numbness (YI=57%), hand clumsiness (YI=50%), walking imbalance (YI=50%), and UE weakness (YI=46%). Questionnaires performed well including mJOA (YI=72%), NDI (YI=63%), and EQ-5D (YI=57%). Physical testing showed best results with UE reflexes (YI=54%), strength in 5 UE muscle groups (YI=53%), Berg Balance scale (YI=50%), self-paced walking velocity (YI=48%), and tandem gait assessment (YI=40%). Hand dexterity, strength dynamometry, and testing of 5 sensory modalities demonstrated poor diagnostic utility. Diagnosis of DCM is challenging, but key symptoms include neck pain, UE weakness, and those captured by the mJOA (particularly UE numbness, hand clumsiness, and walking imbalance). Physical testing of reflexes, manual motor testing, and gait/balance are useful to confirm the diagnosis. These findings offer guidance for clinicians and the development of diagnostic criteria.

**Supplementary Information:**

The online version contains supplementary material available at 10.1038/s41598-025-20928-4.

## Introduction

Degenerative cervical myelopathy (DCM) is a condition caused by compression of the cervical spinal cord, resulting in varying degrees of motor, sensory, and autonomic dysfunction^1^. Its prevalence remains poorly defined, as epidemiological estimates have traditionally relied on operative incidence alone, contributing to the misconception that DCM is a rare condition^2^. However, MRI studies indicate that cervical cord compression is widespread, with up to 59% of adults showing cervical cord compression and 1–5% having undiagnosed DCM^3,4^. Furthermore, the prevalence of DCM is certain to rise as the population ages and more sensitive assessments are developed.

To date, DCM remains a clinical diagnosis corroborated by imaging findings, and no formal diagnostic criteria have been established. Diagnosing DCM is particularly challenging due to several factors, including its variable presentation, a lack of public awareness, and the need for a detailed neurological examination^5,6^. Symptoms can range from mild sensory disturbances to severe motor deficits. Initial symptoms are often intermittent in nature and show a slow, subtle progression, which delays presentation to a health care practitioner^7^. Common symptoms such as hand numbness, gait imbalance, and incoordination are often falsely attributed to age or other conditions^8^. A recent narrative review highlights the low awareness of DCM among health care providers^5^. Moreover, certain clinical signs such as gait ataxia or Hoffman sign may require the expertise of a specialist to detect. As a result, the prevalence of DCM is likely underestimated by studies, such as that by Nouri et al. which estimated the prevalence at under 0.1%^9^. In contrast, a study of elderly patients with hip fractures found 18% had previously undiagnosed DCM^10^.

Addressing the deficiencies in diagnosing DCM requires awareness of the diverse range of symptoms that patients experience and determining the best methods to detect neurological dysfunction. Incorporating subjective patient-reported data alongside reproducible physical tests could reduce provider dependency in making this diagnosis. In this study, we aimed to prospectively evaluate a broad range of symptoms, questionnaires, clinical signs, and physical measurements in a large cohort of DCM and healthy subjects to characterize the sensitivity and specificity, in an effort to contribute toward future development of robust diagnostic criteria.

## Methods

### Study design

This single-institution cross-sectional study was designed as a component of a prospective longitudinal cohort study of patients with DCM and healthy subjects, which received institutional review board (IRB) approval (1723945-1) from University of California, Davis. The study was performed in accordance with relevant ethical guidelines and regulations. Patients who were referred to a spinal neurosurgeon or orthopedic surgeon at University of California, Davis for DCM between August 2021 to December 2024 were evaluated for candidacy. Healthy subjects were recruited by convenience sampling with an emphasis on achieving a balanced range of age and sex to allow for multivariate regression analysis of these variables and to use as a control group in DCM and other neurological conditions. All consecutive DCM patients referred to a neurosurgery or orthopedic spine surgeon that were eligible during the study period were invited to participate, and their enrolment was dependent upon their willingness and availability to complete an extra 1-hour data collection session on either the same day or to return for an additional visit, depending on the availability of the research team. Written informed consent was obtained from all participants prior to enrollment, and data collection was performed in the UC Davis Spinal Cord Injury, Function, and Imaging (SCIFI) Lab and/or the UC Davis Spine Center.

### Eligibility criteria

Inclusion criteria included English-speaking adults aged 18–90 years, while prisoners, pregnant women, and those unable to provide informed consent were excluded. Subjects were also excluded if they had non-degenerative causes of cervical myelopathy such as tumor, infection, cervical trauma, or rheumatoid arthritis. DCM subjects were required to have: 1) **at least one symptom** of upper extremity (UE) numbness/paresthesia, UE incoordination/clumsiness, subjective UE weakness, imbalance when walking, or autonomic dysfunction symptoms including urinary, sexual and bowel dysfunction; 2) **at least one clinical sign** including any hyperreflexia (UE or lower extremity (LE)LE), objective UE weakness, UE sensory loss, or gait ataxia; 3) **imaging evidence of cervical cord compression**, including circumferential compression, indentation, flattening, or torsion (from a lateral disc bulge)^10^ and 4) **the absence of an alternative diagnosis** that more likely explained symptoms. Findings were assessed qualitatively by experienced board certified spine surgeons. Imaging interpretation was performed in the context of a standard spine clinic evaluation and was not blinded to clinical presentation, reflecting real-world diagnostic practice. Subjects were examined for tremor, tongue fasciculations, pronator drift, rigidity, spasticity, rapid alternative movements, and finger-to-nose testing, specifically to identify other neurological conditions. Following data collection, all subjects were reviewed by 2 researchers (K.S. and A.R.M.) to confirm inclusion, and subjects with an uncertain diagnosis were excluded from this analysis. However, we aimed to study a real-world cohort of DCM patients, which often have confounding comorbidities; thus, both DCM patients and healthy subjects were still considered eligible for inclusion if they had minor confounding conditions, (e.g. joint replacement, carpal tunnel syndrome, lumbar radiculopathy), as long as these did not appear to be their primary cause of impairment.

### Data collection

Data collection included medical history, symptoms history, questionnaires, and comprehensive testing of motor, sensory, gait and other neurological functions.

All subjects completed a comprehensive medical history questionnaire to detect potentially confounding comorbidities, including cardiopulmonary, endocrine, inflammatory, neurological, and musculoskeletal disorders, pathologies. A detailed questionnaire was completed with the aid of a research coordinator, who helped interpret questions, focused to determine whether participants experienced any of the following 15 specific symptoms at least once per week: headache, neck pain, arm pain, leg pain, back pain, upper extremity (UE) weakness, lower extremity (LE) weakness, hand incoordination, gait impairment, UE numbness, LE numbness, urinary dysfunction, fecal incontinence, sexual dysfunction, and saddle numbness. Three patient reported outcome measures (PROMs) were collected: the EuroQol 5-Dimension 5-Level (EQ-5D-5L), Quick Disabilities of the Arm, Shoulder, and Hand (QuickDASH), and Neck Disability Index (NDI). The modified Japanese Orthopaedic Association scale (mJOA) was collected, which is a clinician-administered outcome measure (CAOM) where a questionnaire on 4 types of neurological dysfunction is administered, and responses are interpreted by a clinician.

Physical examination and testing were performed by an experienced physician who received detailed training in 4 categories: manual muscle testing, reflexes, sensory test and gait and balance assessment. Deep tendon reflexes were tested with a Queen Square hammer in biceps (Bi), triceps (Tri), brachioradialis (BR), patellar (Pa), and Achilles (Ach) and graded as 0+ (absent), 1+ (diminished or requiring distraction), 2+ (normal), 3+ (brisk), and 4+ (pathological or spreading). Special reflex tests included Hoffmann, Tromner, and Babinski, while inverted brachioradialis (IBR) was recorded if finger flexion was observed during BR testing. Sensory testing included light touch and pain perception in C5-T1 and L2-S1 dermatomes rated as 0 (absent), 1 (altered), or 2 (normal), following the International Standards for Neurological Classification of Spinal Cord Injury (ISNCSCI) methodology^11^. Semmes Weinstein monofilament testing was performed on dermatomes C6, C7, and C8 using the palmar surface of the finger pads, scored as 0 (absent), 1 (insert the actual filament sizes), 2, 3, or 4. Proprioception and vibration (with a 128 Hz tuning fork) were both tested on the index finger and the great toe.

Manual muscle testing (MMT) was performed in 24 muscle groups including: shoulder abduction (SAb), elbow flexion (EF), elbow extension (EE), wrist flexion (WF), wrist extension (WE), finger flexion (FF), finger extension (FE), flexor pollicis longus (FPL), finger abduction (FAb) which consisted of testing the abductor digiti minimi (fifth digit), finger adduction (FAd), first dorsal interossei (1DI), first to fifth digit opposition (Opp), hip flexion (HF), hip extension (HE), hip abduction (HAb), hip adduction (HAd), knee flexion (KF), knee extension (KE), ankle dorsiflexion (AD), ankle eversion (AE), ankle inversion (AI), ankle plantarflexion (AP), extensor hallucis longus (EHL), and flexor hallucis longus (FHL). Muscle strength was graded 0 to 5 using the Medical Research Council (MRC) scale. Grip, pinch (1^st^ and 2^nd^ digits), opposition (1^st^ and 5^th^ digits), and adduction (2^nd^ and 3^rd^ digits) were measured using Jamar dynamometers as the average of 3 trials.

Balance was assessed using an abbreviated (9-item) version of the Berg Balance scaleClick or tap here to enter text^11^, including: standing with feet together (Romberg with eyes open test), Romberg with eyes closed, reaching forward, picking up an object from the ground, turning to look over each shoulder, turning 360, alternating stepping, tandem stance (heel-to-toe), and single-leg stance. Quantitative gait and balance testing was also performed using Protokinetics Movement Analysis Software (PKMAS) on a Zeno electronic pressure mat (ProtoKinetics LLC, Havertown, PA). Tasks included self-paced walking, fast-paced walking, Romberg eyes-open, Romberg eyes-closed, tandem gait, tandem stance, and single-leg stance (performed each foot)^13^. Each task was manually scored by an researcher by counting stumbles (side-stepping or touching a wall/researcher for support) and wobbles (major shifts of the trunk to maintain balance), and this scoring scheme was subsequently simplified into a 6-level ordinal scale. Electronic parameters for walking tests included velocity, stride length, stride width, cadence, single-stance time, double-stance time, stability ratio (single-stance time/double-stance time), and gait variability index (GVI).

### Statistical analysis

Data were analyzed with R 4.4.1. Continuous data were reported as mean ± standard deviation (SD). Continuous variables were compared between DCM patients and healthy controls using two-tailed t-tests. Categorical and binary variables were compared using Chi-square tests. Results were noted as statistically significant for p<0.05 and corrected for multiple comparisons using the Benjamini–Hochberg to control the false discovery rate. Statistical values for demographics were not corrected given that they were not considered hypothesis. In this study, uncorrected p < 0.05 (*) was used to identify a trend, corrected p < 0.05 (**) was used to denote statistically significant, and correct p < 0.001 (***) was used to delineate highly significant results. Sensitivity (SE), specificity (SP), and Youden’s Index (YI = SE + SP - 1) were calculated in R. Receiver operating characteristic (ROC) curves were generated in R for numeric variables to calculate area under the curve (AUC) and automatically generate optimized thresholds to maximize YI. All variables were ranked by YI for the purpose of comparing diagnostic utility. Symptoms were coded as present or absent, with questions standardized to refer to symptom occurrence over the past week, differing from tools such as EQ-5D and NRS that measure current symptoms.

## Results

### Demographics and patient characteristics

A total of 162 DCM patients and 128 healthy subjects were enrolled. After application of eligibility criteria, 139 DCM and 108 healthy subjects were included in the final analysis. DCM subjects had elevated BMI, weight and had a preponderance of white race, while other patient characteristics were similar (BMI: two-tailed t-test, *p* = 0.04; weight: two-tailed t-test, *p* = 0.03; race: Chi-square test, *p* = 0.23; age: two-tailed t-test, *p* = 0.16; sex: Chi-square test, *p* = 0.57; height: two-tailed t-test, *p* = 0.80; hand dominance: Chi-square test, *p* = 0.94) (Table 1).

**Table 1:** Participant demographics and baseline clinical characteristics.

**Measure**	**DCM (n=139)**	**Healthy (n=108)**	**P value**
Age, mean (SD)	60.8 (14.0)	57.9 (16.9)	0.08
BMI, mean (SD)	29.2 (7.7)	27.3 (6.6)	0.04
Height (inches) mean (SD)	65.6 (4.1)	65.4 (4.1)	0.87
Weight (lbs) mean (SD)	178.8 (47.1)	166.4 (42.7)	0.04
**Sex, no (%)**			0.57
Male	47 (34)	32 (30)	
Female	92 (66)	76 (70)	
**Race, no (%)**			0.23
White (Caucasian/European)	110 (79)	79 (57)	
Hispanic or Latino	9 (6)	14 (10)	
Asian	6 (4)	8 (6)	
Pacific Islander	1 (1)	2 (1)	
Black or African	6 (4)	4 (3)	
Native American	2 (1)	0 (0)	
Other/Unspecified	5 (4)	1 (1)	
**Hand dominance, no (%)**			0.94
Right	122 (88)	96 (89)	
Left	12 (9)	8 (7)	
Both	5 (4)	4 (4)	

Values are presented as mean (SD) for continuous variables and n (%) for categorical variables. P values were calculated using two-tailed t-tests for continuous variables and Chi-square tests for categorical variables and were not corrected for multiple comparisons. SD indicates variability within groups.

*Abbreviations: Degenerative Cervical Myelopathy (DCM), Body Mass Index (BMI), Standard Deviation (SD), Number (No.)*.

*Data was missing for 1 participant in the healthy control group for hand dominance.*

A total of 216/281 (77%) outcome measures showed significant differences between DCM and healthy subjects (p < 0.05, two-tailed tests for continuous variables; Chi-square for categorical variables). These differences spanned across clinical signs, symptoms, and functional assessments, highlighting distinct profiles between the two groups.

### Symptoms

**Figure 1:**
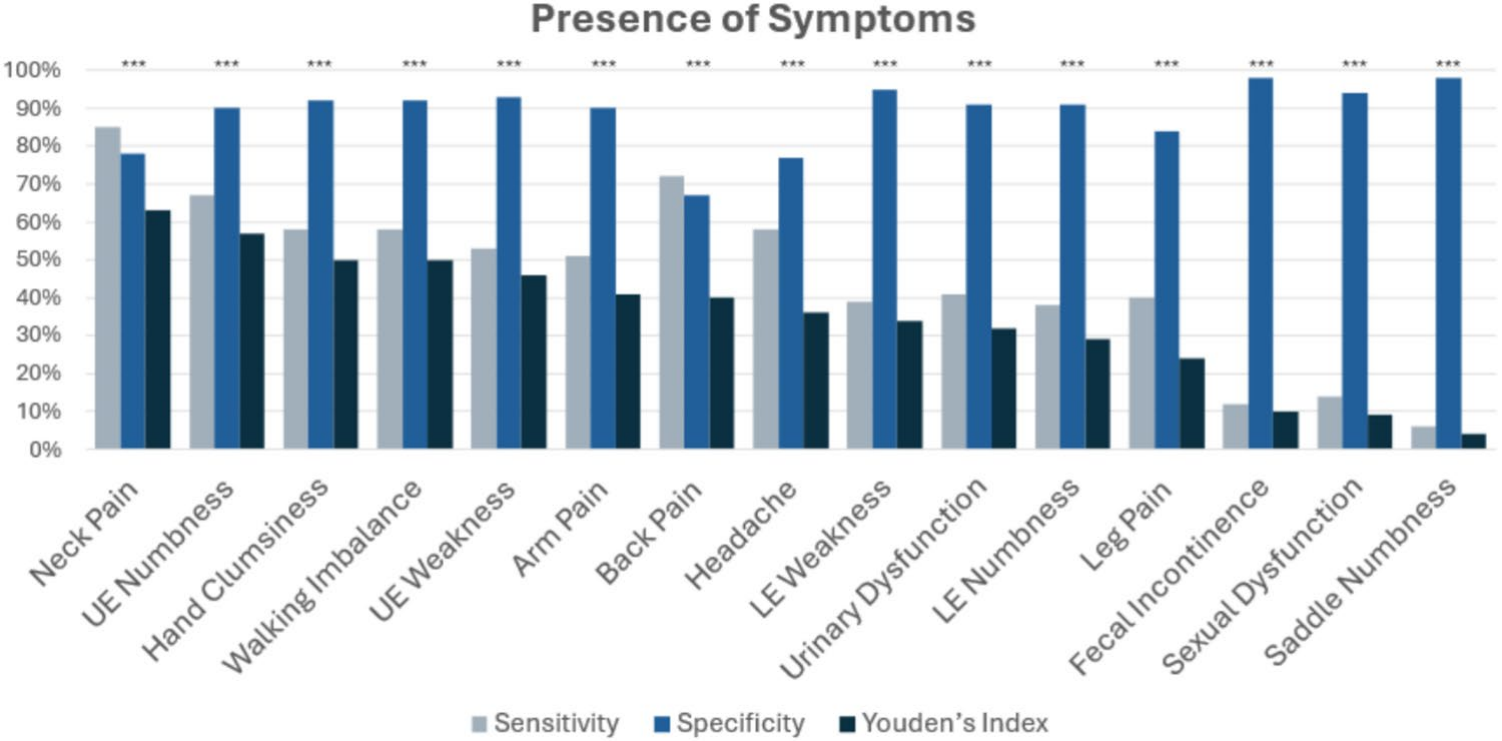
Diagnostic accuracy of symptoms between healthy controls (HCS) and DCM patients. The plots are based on the data reported in the results section. DCM with n=139; healthy with n=108. Neck Pain (SE=85%, SP=78%, YI=63%); UE numbness (SE=67%, SP=90%, YI=57%); hand incoordination (SE=58%, SP=92%, YI=50%); gait imbalance (SE=58%, SP=92%, YI=50%); UE weakness (SE=53%, SP=93%, YI =46%); arm pain (SE=51%, SP=90%, YI=41%); back pain (SE=72%, SP=67%, YI=40%); headache SE=58%, SP77%, YI=36%); lower extremity weakness (SE=39%, SP=95%, YI=34%); urinary dysfunction (SE= 41%, SP=91%, YI=32%) lower extremity numbness (SE=38%, SP=91%, YI=29%); leg pain (SE=40%, SP=84%, YI=24%);fecal incontinence (SE=12%, SP=98%, YI=10%); sexual dysfunction (SE=14%, SP=94%, YI=9%); SE=58%, SP, YI=%); saddle numbness (SE=6%, SP=98%, YI=4%). ‘*’ was used to denote a trend (uncorrected p < 0.05), ‘**’ represents statistical significance (corrected p < 0.05), and ‘***’ indicates a highly significant result (corrected p < 0.001).

Neck pain was the most distinguishing symptom between DCM and healthy subjects (n=139 DCM, n=108 healthy; SE=85%, SP=78%, YI=63%), followed by UE numbness (SE=67%, SP=90%, YI=57%), hand incoordination (SE=58%, SP=92%, YI=50%), gait imbalance (SE=58%, SP=92%, YI=50%), UE weakness (SE=53%, SP=93%, YI =46%), arm pain (SE=51%, SP=90%, YI=41%), and back pain (SE=72%, SP=67%, YI=40%). Urinary dysfunction (SE= 41%, SP=91%, YI=32%) had a lower discriminatory value. (Figure 1 and Supplementary Table 1).

### Questionnaires

Total mJOA score showed good performance (n=139 DCM, n=108 healthy; SE=82%, SP=90%, YI=72%) at a threshold of <16 to predict a diagnosis of DCM (Table 2). When mJOA subscores were assessed individually, the UE sensory component had the highest sensitivity and discrimination (SE=77%, SP=85%, YI=62%). The UE motor subscore had the highest specificity but lower sensitivity (SE=64%, SP=93%, YI=57%). The total NDI score also performed well (SE=78%, SP=85%, YI=63%). NDI questions revealed that DCM has specific impacts on lifting (SE=72%, SP=89%, YI=61%), driving (SE=68%, SP=91%, YI=59%), pain intensity (SE=80%, SP=78%, YI=58%), work (SE=64%, SP=94%, YI=58%), and recreational activities (SE=68%, SP=88%, YI=56%). The total EQ-5D-5L score demonstrated moderate diagnostic accuracy (SE=74%, SP=83%, YI=57%), with notable impacts on usual activities (SE=64%, SP=93%, YI=57%), pain/discomfort (SE=65%, SP=90%, YI=55%), and mobility (SE=62%, SP=87%, YI=49%). EQ-VAS had slightly diminished performance compared with EQ-5D-5L (SE=70%, SP=83%, YI=53%).

**Table 2:** Diagnostic accuracy of questionnaires.

Questionnaire	Component	Sensitivity	Specificity	Youden’s index	AUC	Threshold	P value
mJOA	UE Sensory	77%	85%	62%	83%	< 3	<0.001
	LE Motor	72%	89%	61%	82%	< 7	<0.001
	UE Motor	64%	93%	57%	79%	< 5	<0.001
	Urinary	45%	90%	35%	68%	< 3	<0.001
	**Total**	**82%**	**90%**	**72%**	**93%**	**< 16**	<0.001
NDI	Lifting	72%	89%	61%	83%	< 1	<0.001
	Driving	68%	91%	59%	81%	< 1	<0.001
	Pain Intensity	80%	78%	58%	81%	< 1	<0.001
	Work	64%	94%	58%	80%	< 1	<0.001
	Recreation	68%	88%	56%	80%	< 1	<0.001
	Reading	70%	82%	52%	80%	< 1	<0.001
	Sleeping	64%	82%	46%	74%	< 1	<0.001
	Concentration	61%	84%	45%	73%	< 1	<0.001
	Personal care	42%	96%	38%	70%	< 1	<0.001
	Headaches	64%	72%	36%	70%	< 1	<0.001
	**Total**	**78%**	**85%**	**63%**	**87%**	**< 6**	<0.001
EQ-5D-5L	Usual activities	64%	93%	57%	79%	< 2	<0.001
	Pain/Discomfort	65%	90%	55%	84%	< 3	<0.001
	Mobility	62%	87%	49%	74%	< 2	<0.001
	Self-care	43%	96%	39%	70%	< 2	<0.001
	Anxiety and depression	28%	92%	20%	61%	< 3	<0.001
	**Total**	**74%**	**83%**	**57%**	**87%**	**< 8**	<0.001
EQ-VAS		**70%**	**83%**	**53%**	**78%**	**< 78**	<0.001

Values reflect the diagnostic performance of questionnaire components using ROC analysis. Sensitivity, specificity, Youden’s Index, area under the curve (AUC), and optimal classification thresholds were determined for each metric. Higher AUC and Youden’s Index values indicate better discriminative ability. P values displayed were corrected for multiple comparisons using Benjamini-Hochberg procedure.

*Abbreviations: modified Japanese Orthopaedic Association Score (mJOA), Upper extremity (UE), Lower extremity (LE), Neck Disability Index (NDI), EuroQol Five-Dimension Five-Level (EQ-5D-5L), EuroQol Visual Analogue Scale (EQ-VAS), degenerative cervical myelopathy (DCM), healthy control (HCS).*

*Data were missing for the EQ-5D pain/discomfort subscore and all NDI subscores for one HCS. There were no missing data for DCM patients.*

### Muscle strength

**Figure 2:**
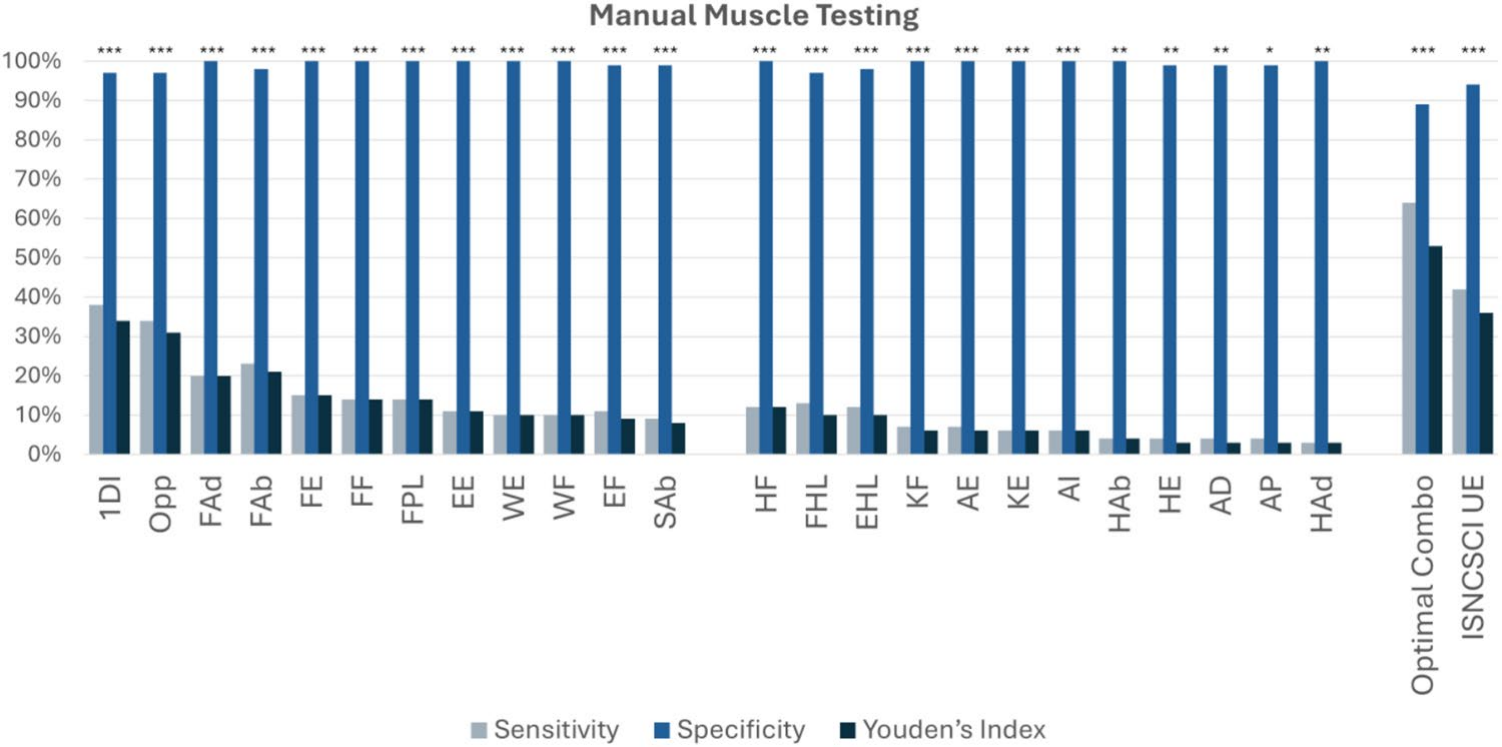
Manual Motor Testing of Upper and Lower Extremities. The plots are based on the data reported in the results section and Tables 3 and 4. DCM with n=139; healthy with n=108. Details of the muscle groups and functions assessed are listed in Supplementary Table 2. Optimal Combo: EF, FE, FF, 1DI, Opp. ISNCSCI UE: EF, WE, EE, FF, Fab. ‘*’ was used to denote a trend (uncorrected p < 0.05), ‘**’ represents statistical significance (corrected p < 0.05), and ‘***’ indicates a highly significant results (corrected p < 0.001). *Abbreviations: Elbow Flexion (EF), Finger Extension (FE), Finger Flexion (FF), First Dorsal Interosseous (1DI), Opponens (Opp), International Standards for Neurological Classification of Spinal Cord Injury (ISNCSCI), Upper Extremity (UE).*

Among the UE muscle groups tested, hand intrinsics showed the highest discriminatory values including 1DI (n=139 DCM, n=108 healthy; SE=38%, SP=97%, YI=34%), Opp (SE=34%, SP=97%, YI=31%), FAd (SE=20%, SP=100%, YI=20%), and FAb (SE=23%, SP=98%, YI=21%) (Table 3, Table 4, and Figure 2). Other finger control muscles also demonstrated moderate sensitivity and high specificity including FE (SE=15%, SP=100%, YI=15%), FF (SE=14%, SP=100%, YI=14%), and FPL (SE=14%, SP=100%, YI=14%). Proximal muscles involving C5 and C6 myotomes exhibited lower sensitivity, including EF (SE=11%, SP=99%, YI=9%) and SAb (SE=9%, SP=99%, YI=8%). LE muscle testing generally showed poor diagnostic performance, with weakly positive results in HF (SE=12%, SP=100%, YI=12%), FHL (SE=13%, SP=97%, YI=10%), and EHL (SE=12%, SP=98%, YI=10%) (Table 4). Diagnostic performance improved when muscle groups were combined, including using the ISNCSCI UE motor examination (SE=42%, SP=94%, YI=36%), and using an optimized combination of EF, FE, FF, 1DI, and Opp (SE=64%, SP=89%, YI=53%). Strength dynamometry demonstrated poor discrimination, including 1–2 pinch strength (SE=64%, SP=64%, YI=28%) and hand grip strength (SE=43%, SP=74%, YI=18%) (Table 5 and Table 6).

**Table 3:** Diagnostic accuracy of manual muscle testing in the upper extremity.

Manual motor testing	Dermatome	Sensitivity	Specificity	Youden’s index	AUC	Threshold	P value
1DI	T1	38%	97%	34%	67%	<5	<0.001
Opp	T1	34%	97%	31%	65%	<5	<0.001
FAd	T1	20%	100%	20%	60%	<5	<0.001
FAb	T1	23%	98%	21%	60%	<5	<0.001
FE	C7	15%	100%	15%	57%	<5	<0.001
FF	C8	14%	100%	14%	57%	<5	<0.001
FPL	C8	14%	100%	14%	57%	<5	<0.001
EE	C7	11%	100%	11%	55%	<5	<0.001
WE	C6/7	10%	100%	10%	55%	<5	<0.001
WF	C8	10%	100%	10%	55%	<5	<0.001
EF	C5/6	11%	99%	9%	55%	<5	<0.001
SAb	C5	9%	99%	8%	54%	<5	<0.001
ISNCSCI UE	C5-T1	42%	94%	36%	69%	<50	<0.001
Optimized combination (EF, FE, FF, 1DI, Opp)	C5-T1	64%	89%	53%	79%	<10	<0.001

Sensitivity, specificity, Youden’s Index, and AUC were calculated using ROC analysis for each manual motor test related to cervical myelopathy detection. The optimal classification thresholds indicate the point below which patients were classified as likely to have DCM. The optimized combination represents the best-performing subset of motor tests based on the highest discriminative ability. P values displayed were corrected for multiple comparisons using Benjamini-Hochberg procedure.

*Abbreviations: First Dorsal Interosseous (1DI), Opponens Pollicis (Opp), Finger Adduction (Fad), Finger Abduction (Fab), Finger Extensors (FE), Finger Flexors (FF), Flexor Pollicis Longus (FPL), Extrinsic Extensors (EE), Wrist Extensors (WE), Wrist Flexors (WF), Elbow Flexors (EF), Shoulder Abductors (Sab), International Standards for Neurological Classification of Spinal Cord Injury (ISNCSCI) for Upper Extremity (ISNCSCI UE).*

*There were no missing data.*

**Table 4:** Diagnostic accuracy of manual muscle testing in the lower extremity.

Manual muscle testing	Dermatome	Sensitivity	Specificity	Youden’s index	AUC	Threshold	P value
Hip flexion	L1/L2/L3	12%	100%	12%	56%	<5	<0.001
Flexor hallucis longus	L5/S1	13%	97%	10%	55%	<5	<0.001
Extensor hallucis longus	L5/S1	12%	98%	10%	55%	<5	<0.001
Knee flexion	S1	7%	100%	6%	53%	<5	<0.001
Ankle eversion	L4-S1	7%	100%	6%	53%	<5	<0.001
Knee extension	L3/L4	6%	100%	6%	53%	<5	<0.001
Ankle inversion	L2-L4	6%	100%	6%	53%	<5	<0.001
Hip abduction	L4-S1	4%	100%	4%	52%	<5	<0.01
Hip extension	L4-S1	4%	99%	3%	52%	<5	<0.01
Ankle dorsiflexion	S1/S2	4%	99%	3%	51%	<5	<0.05
Ankle plantarflexion	L2-L4	4%	99%	3%	51%	<5	0.06
Hip adduction	L2-L3	3%	100%	3%	51%	<5	0.03

Sensitivity, specificity, Youden’s Index, and AUC were calculated using ROC analysis for each lower extremity manual muscle test. Thresholds reflect the manual strength score below which patients were considered positive for DCM-related impairment. P values displayed were corrected for multiple comparisons using Benjamini-Hochberg procedure.

*Abbreviations: Hip flexion (HF), Flexor Hallucis Longus (FHL), Extensor Hallucis Longus (EHL), Knee extension (quadriceps)(KE), Knee flexion (hamstrings)(KF), Hip adduction (HAd), Hip abduction (HAb), Ankle dorsiflexion (AD), Ankle plantarflexion (AP), Hip extension (gluteus maximus) (HE), Ankle eversion (AE), Ankle inversion (AI)*.

*There were no missing data.*

**Table 5:** Diagnostic accuracy of strength dynamometry.

Strength dynamometry	Sensitivity	Specificity	Youden’s index	AUC	Threshold (kg)	P value
Thumb-finger (1–2) pinch	64%	64%	28%	68%	< 6	<0.001
Hand grip	43%	74%	18%	61%	< 19	<0.001
Finger-finger (2–3) pinch	45%	73%	18%	60%	< 1.2	<0.001
Thumb-finger (1–5) pinch	27%	91%	18%	59%	< 2	<0.001

ROC analysis was used to evaluate the diagnostic performance of upper extremity strength dynamometry tests for detecting cervical myelopathy. The optimal threshold represents the force (in kilograms) below which patients were classified as impaired. Youden’s Index and AUC indicate overall discriminative performance; values closer to 1 reflect higher accuracy. P values displayed were corrected for multiple comparisons using Benjamini-Hochberg procedure.

*Missing data: Hand grip in 1 DCM patient and 4 HCS; 1–2 pinch in 4 DCM patients and 4 HCS; 1–5 pinch in 5 DCM patients and 4 HCS; 2–3 pinch in 4 DCM patients and 4 HCS.*

**Table 6:** Diagnostic accuracy of GRASSP.

Hand dexterity	Sensitivity	Specificity	Youden’s index	AUC	Threshold	P value
GRASSP-M time	52%	80%	31%	68%	>54	<0.001
GRASSP-M completion subscore	21%	96%	16%	58%	< 2	<0.001
GRASSP-M drops subscore	37%	69%	6%	54%	< 3	0,10
GRASSP-M grasp subScore	31%	74%	5%	53%	< 4	0.01
GRASSP-M total	**27%**	**88%**	**14%**	**57%**	**< 8**	0.17

Values depict the diagnostics performance of the GRASSP-M hand dexterity assessment components for detecting cervical myelopathy. Sensitivity, specificity, and AUC values were derived from ROC analysis. Thresholds indicate the point at which performance suggests impairment. P values displayed were corrected for multiple comparisons using Benjamini-Hochberg procedure.

*Abbreviations: Graded Redefined Assessment of Strength, Sensibility, and Prehension (GRASSP)*.

*There were no missing data.*

### Sensation

Sensory testing showed similar overall results across different sensory modalities when using total scores, including monofilaments (n=139 DCM, n=108 healthy; SE=32%, SP=93%, YI=25%), light touch (LT) (SE=26%, SP=98%, YI=24%), pin prick (PP) (SE=26%, SP=98%, YI=24%), vibration (SE=38%, SP=84%, YI=22%), and proprioception (SE=29%, SP=91%, YI=20%) (Table 7). Vibration and proprioception demonstrated higher sensitivity in the great toe (SE=27%, 22%, respectively) compared to the index finger (SE=5%, 10%). Testing of specific dermatomes showed highest sensitivity in C6 for monofilaments (SE=17%), while C7 was highest for PP (SE=12%), and C5, C6, and C7 were highest for LT (SE=8–9%).

**Table 7:** Diagnostic accuracy of sensory testing.

Sensory modality	Location	Sensitivity	Specificity	Youden index	AUC	Threshold	P value
Light touch	C5	9%	100%	8%	54%	< 2	<0.001
	C6	8%	100%	8%	54%	< 2	<0.001
	C7	8%	100%	8%	54%	< 2	<0.001
	C8	7%	100%	6%	53%	< 2	<0.001
	T1	6%	100%	6%	53%	< 2	<0.001
	**Total**	**26%**	**98%**	**24%**	**62%**	**< 20**	<0.001
Pin prick	C5	10%	99%	9%	54%	< 2	<0.001
	C6	11%	99%	10%	55%	< 2	<0.001
	C7	12%	99%	11%	55%	< 2	<0.001
	C8	9%	99%	8%	54%	< 2	0.001
	T1	6%	99%	5%	53%	< 2	0.02
	**Total**	**26%**	**98%**	**24%**	**62%**	**< 20**	<0.001
Monofilaments	C6	17%	97%	13%	57%	< 4	<0.001
	C7	10%	98%	8%	54%	< 4	<0.001
	C8	9%	98%	7%	54%	< 4	0.002
	**Total**	**32%**	**93%**	**25%**	**63%**	**< 24**	<0.001
Vibration	Index Finger	5%	99%	4%	52%	< 1	0.01
	Great Toe	27%	89%	16%	58%	< 1	<0.001
	**Total**	**38%**	**84%**	**22%**	**60%**	**< 4**	<0.001
Proprioception	Index Finger	10%	95%	4%	52%	< 2	0.001
	Great Toe	22%	92%	14%	57%	< 2	<0.001
	**Total**	**29%**	**91%**	**20%**	**60%**	**< 8**	<0.001

Sensitivity, specificity, Youden’s Index, and AUC values were derived using ROC analysis for each sensory test modality at specified dermatomes or anatomical sites. Individual tests are analyzed per side, while total results are summed across both sides. Thresholds represent the lowest score or value indicative of sensory impairment. Low sensitivity across modalities suggests limited standalone diagnostic power. P values displayed were corrected for multiple comparisons using Benjamini-Hochberg procedure.

*There were no missing data.*

### Reflexes

**Figure 3:**
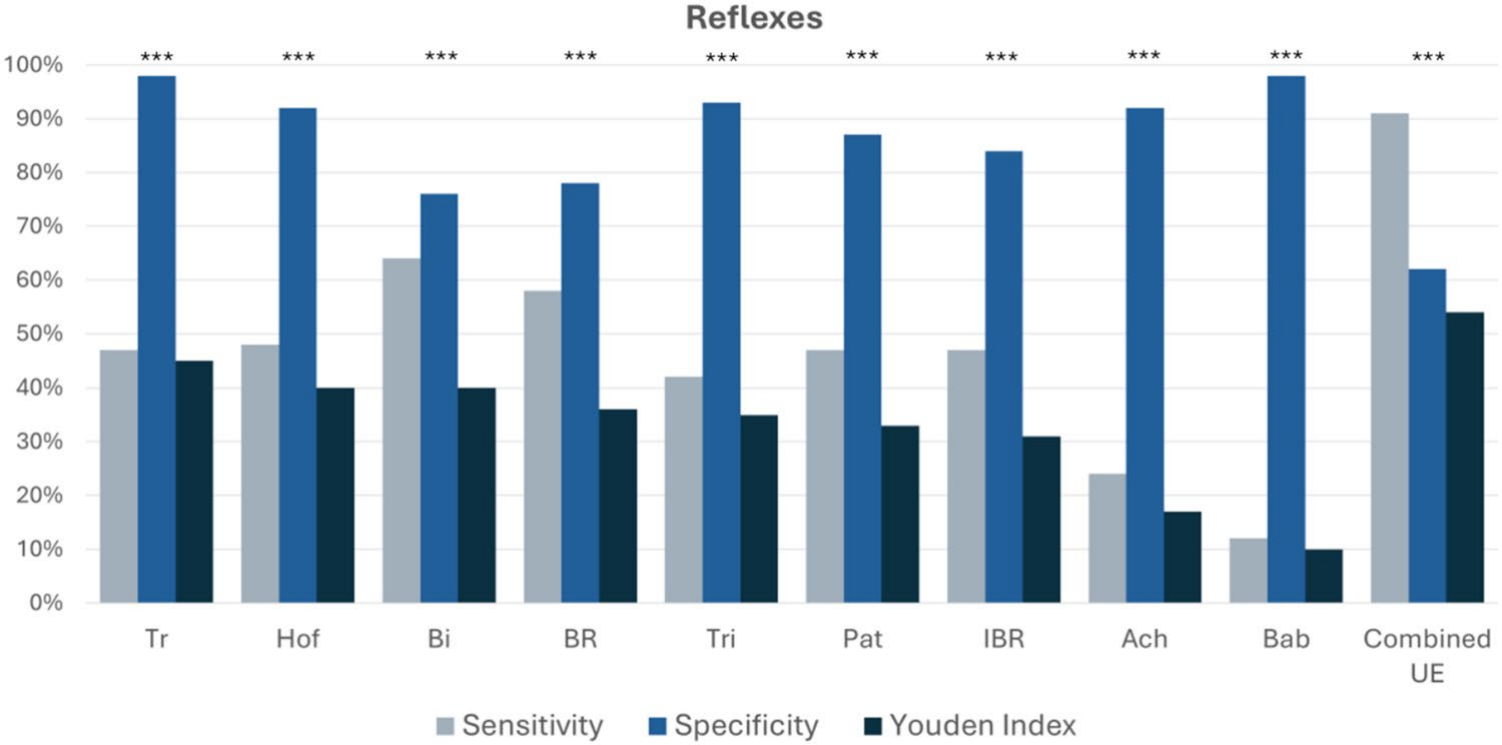
Diagnostic Accuracy of Deep Tendon Reflexes and Special Reflex Tests. The plots are based on the data reported in the results section and Table 8. Combined UE reflexes included Bi, Tri, BR, IBR, and Tr. ‘*’ was used to denote a trend (uncorrected p < 0.05), ‘**’ represents statistical significance (corrected p < 0.05), and ‘***’ indicates a highly significant results (corrected p < 0.001). *Abbreviations: Tromner reflex (Tr), Hoffmann (Hof), Biceps reflex (Bi), triceps reflex (Tri), Brachioradialis reflex (BR), Inverted brachioradialis reflex (IBR), Achilles (Ach), Babinski (Bab), Upper Extremity (UE).*

Tromner (n= 139 DCM, n=108 healthy; SE=47%, SP=98%, YI=45%), and Hoffmann (SE=48%, SP=92%, YI=40%) reflexes demonstrated the highest discriminatory value among the reflex tests. Deep tendon reflexes of the UEs, including biceps (SE=64%, SP=76%, YI=40%), brachioradialis (SE=58%, SP=78%, YI=36%), and triceps (SE=42%, SP=93%, YI=35%) demonstrated stronger performance compared to those in the LEs, including patellar (SE=47%, SP=87%, YI=33%) and Achilles (SE=24%, SP=92%, YI=17%). Babinski test sign demonstrated poor sensitivity (SE=12%, SP=98%, YI=10%) (Table 8 and Figure 3).

**Table 8:** Diagnostic accuracy of reflexes and special reflex tests.

Reflexes	Sensitivity	Specificity	Youden index	AUC	Threshold	P value
Tromner	47%	98%	45%	72%	Presence	<0.001
Hoffmannn	48%	92%	40%	70%	Presence	<0.001
Biceps	64%	76%	40%	70%	>2	<0.001
Brachioradialis	58%	78%	36%	70%	>2	<0.001
Triceps	42%	93%	35%	71%	>2	<0.001
Patellar	47%	87%	33%	67%	>2	<0.001
Inverted brachioradialis	47%	84%	31%	66%	Presence	<0.001
Achilles	24%	92%	17%	56%	>2	<0.001
Babinski	12%	98%	10%	55%	Presence	<0.001
Combined UE (Bi, Tri, BR, IBR, Tr)	91%	62%	54%	81%	Any abnormal	<0.001

Reflex-based screening tests were evaluated for their diagnostic performance in identifying cervical myelopathy. Sensitivity, specificity, Youden’s Index, area under the curve (AUC), and optimal classification thresholds were determined for each screening test. “Presence” indicates the clinical presence of the reflex. Combined reflex scores increase overall sensitivity, demonstrating the benefit of interpreting them together. Reflexes were graded from 0 to 4 based on Medical Research Council Scale and were considered abnormal if 3 or greater. Hoffman, Tromner, and Babinski were considered abnormal when present. Additionally, the combined UE score was considered abnormal if any individual reflex was abnormal in either limb. P values displayed were corrected for multiple comparisons using Benjamini-Hochberg procedure.

*Abbreviations: Biceps reflex (Bi), triceps reflex (Tri), Brachioradialis reflex (BR), Inverted brachioradialis reflex (IBR), Tromner reflex (Tr)*.

*There were no missing data.*

### Gait and balance testing

**Figure 4:**
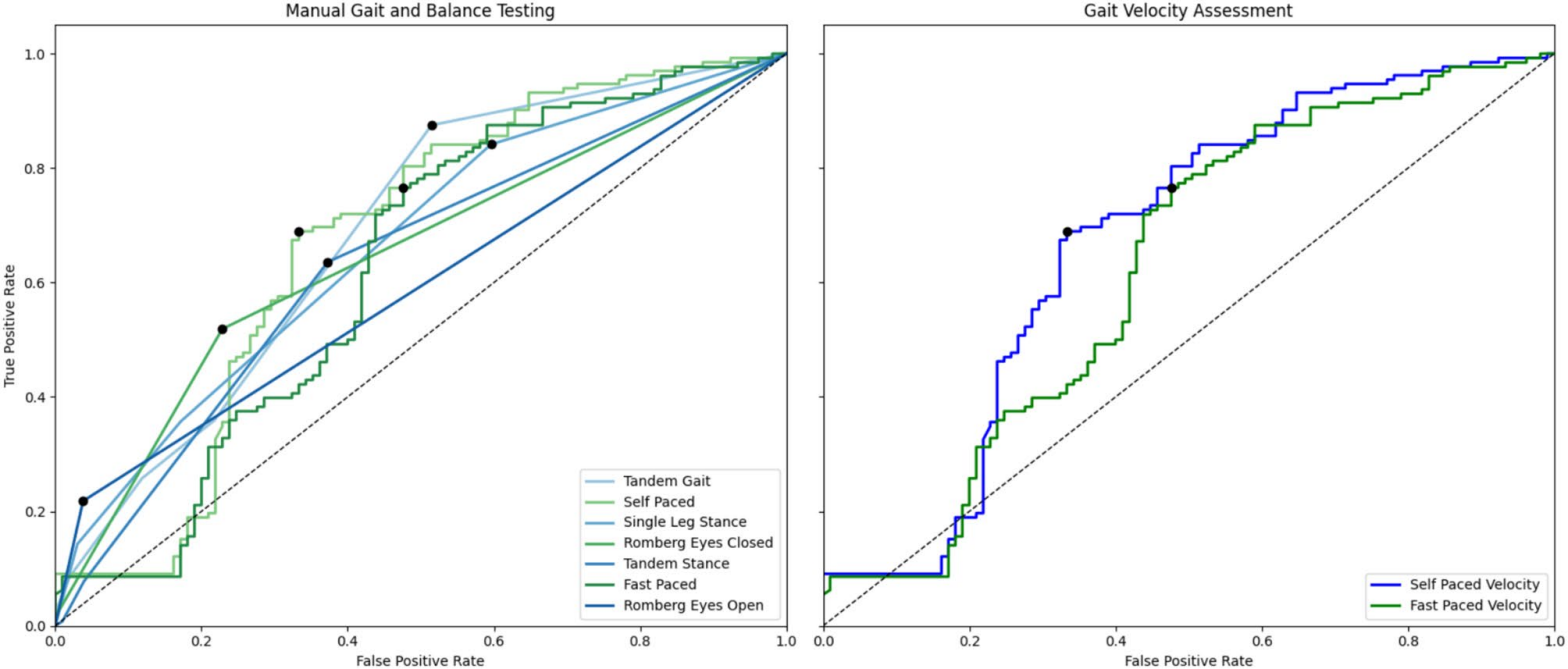
Receiver-operating characteristic (ROC) curves for gait and balance measures. ROC curves were calculated for all numeric variables, and optimal thresholds were automatically selected that optimized Youden’s Index. Measures included scored assessment of self-paced walk, fast-paced walk, tandem gait, tandem stance, Romberg test (eyes open and closed), standing on one foot, and self/fast paced velocity. *Abbreviations: Area under the curve (AUC)*.

Gait assessments demonstrated good discrimination between DCM and healthy subjects. Tandem gait (n=139 DCM, n=108 healthy; SE=87%, SP=53%, YI=40%), abbreviated Berg Balance (SE=67%, SP=83%, YI=50%), and self-paced walking velocity (SE=69%, SP=79%, YI=48%) showed the highest performance (Figure 4). Tandem stance (SE=63%, SP=61%, YI=25%) and single-leg stance (SE=84%, SP=40%, YI=24%) showed moderate diagnostic value (Table 9). Several of the Berg Balance components showed similar results to scored balance assessments, including single-leg stance, tandem stance, and Romberg tests (Table 10 and Table 11).

**Table 9:** Diagnostic accuracy of manual gait and balance measures.

	Sensitivity	Specificity	Youden index	AUC	Threshold	P value
Self-paced velocity	69%	79%	48%	77%	< 100	<0.001
Fast-paced velocity	73%	66%	40%	72%	< 150	<0.001
Tandem gait	87%	53%	40%	72%	< 8	<0.001
Single-leg stance	84%	40%	24%	67%	< 8	<0.001
Tandem stance	63%	61%	25%	63%	< 9	0.06
Romberg eyes closed	52%	70%	22%	61%	< 9	0.06
Romberg eyes open	21%	97%	18%	59%	< 10	0.03

Values depict the diagnostic performance of various gait and balance assessments in identifying functional impairments. ROC analysis was used to calculate sensitivity, specificity, Youden’s Index, and AUC values. Thresholds represent the cutoff scores below which impairment was suspected. P values displayed were corrected for multiple comparisons using Benjamini-Hochberg procedure.

**Table 10:** Diagnostic accuracy of abbreviated 9-item berg balance.

Berg balance component	Sensitivity	Specificity	Youden index	AUC	Threshold	P value
Single-leg stance	50%	86%	37%	67%	< 4	0.001
Turning over shoulders	36%	94%	30%	65%	< 4	<0.001
Tandem stance	35%	88%	23%	61%	< 4	0.06
Foot on step x8	24%	99%	23%	62%	< 4	<0.001
Pick up object	18%	97%	15%	58%	< 4	<0.001
Reaching	14%	99%	13%	57%	< 4	<0.001
Romberg eyes closed	13%	98%	11%	56%	< 4	0.002
Romberg eyes open	12%	97%	9%	54%	< 4	0.009
Turn 360°	6%	100%	6%	53%	< 4	0.02
Total	67%	83%	50%	75%	< 36	<0.001

Values depict the diagnostic performance metrics for individual components and total score of the Berg Balance Scale in detecting balance impairment. Sensitivity, specificity, Youden’s Index, and AUC were calculated using ROC analysis for each item. Thresholds indicate cutoff scores below which impairment was suggested. P values displayed were corrected for multiple comparisons using Benjamini-Hochberg procedure.

**Table 11:** Diagnostic accuracy of quantitative gait analysis.

		Sensitivity	Specificity	Youden index	AUC	Threshold	P value
Self-paced Walking	Velocity	69%	79%	48%	77%	< 100	<0.001
	Double support time	82%	65%	48%	78%	< 0.3	<0.001
	Stability ratio	77%	68%	46%	77%	< 0.7	<0.001
	Stride length	72%	71%	44%	76%	< 117	<0.001
	Gait variability index (GVI)	62%	80%	42%	77%	< 118	<0.001
	Cadence	68%	71%	39%	72%	< 108	<0.001
	Stride width	26%	89%	15%	57%	< 13	0.08
	Single support time	16%	94%	10%	51%	< 0.4	0.11
Fast-paced walking	Stability ratio	82%	59%	41%	74%	< 0.5	<0.001
	Velocity	73%	66%	40%	72%	< 156	<0.001
	Double support time	85%	54%	39%	74%	< 0.21	<0.001
	Stride length	73%	60%	33%	71%	< 135	<0.001
	Gait variability index (GVI)	67%	64%	31%	69%	< 116	<0.001
	Cadence	47%	79%	25%	66%	< 126	<0.001
	Stride width	29%	88%	17%	57%	< 12	0.04
	Single support time	41%	64%	6%	48%	< 0.3	0.13

Analysis was performed on an electronic pressure mat and analyzed using semi-automated analysis. Values depict the diagnostic performance metrics for gait parameters at self-paced and fast-paced walking speeds. Sensitivity and specificity indicate the ability to correctly identify impairment or non-impairment. Youden’s Index summarizes the overall accuracy. AUC (Area Under the Curve) reflects the discriminative ability of each parameter. Thresholds indicate cutoff values used to define impairment. P values displayed were corrected for multiple comparisons using Benjamini-Hochberg procedure.

## Discussion

In the current study, we report the diagnostic accuracy of a broad range of symptoms, questionnaires, clinical signs, and physical tests in a large cohort of DCM patients and healthy subjects. Existing literature on the diagnosis of DCM is scant, with only 3 small studies investigating the diagnostic accuracy of symptoms, and 11 small and/or retrospective studies reporting signs^12^. In this context, our study represents the largest prospective study on diagnostic accuracy of both symptoms and signs. Furthermore, our study provides a number of novel insights due to the comprehensive nature of our assessments, which required approximately 1 hour per session. Our data indicates that the most important symptoms to differentiate between DCM patients and healthy subjects are neck pain, UE numbness, hand clumsiness, walking imbalance, UE weakness (e.g. difficulty opening jars), arm pain, and headache (Figure 1). Furthermore, our findings suggest that the most useful physical tests include UE manual muscle testing (particularly of the hand intrinsics), UE reflex testing, and both gait and balance testing (e.g. tandem gait, timed walking). These results help to refine our understanding of DCM and simplify its assessment and diagnosis, which we hope will lead to the development robust diagnostic criteria and contributed to improved clinical management of DCM in the near future.

Surprisingly, we found that the symptom with highest diagnostic accuracy was neck pain, with high sensitivity (85%) and moderate specificity (78%). This is of particular importance, as neck pain is frequently dismissed by clinicians as non-neurological and is not included in the JOA or mJOA tools, which are the most common outcome measures used in DCM^13^. However, the frequency of neck pain in our DCM cohort is substantially higher than 51% reported in a recent review and meta-analysis^12^, a difference that may be related to the population studied and how the question was phrased (pain over the past week). Furthermore, neck pain has limited specificity for DCM, as several other conditions can cause this symptom. Thus, further research is needed to determine the importance of neck pain, such as determining how often patients presenting solely with this symptom actually have myelopathy. However, it is increasingly recognized that neck pain is often the first symptom of DCM, and its presence should at least raise concern that DCM is a possibility, leading to an appropriate examination and, based on any positive findings, a low threshold to investigate further with MRI. We found that UE numbness, hand incoordination, and walking imbalance were moderately sensitive (67%, 58%, and 58%, respectively) and highly specific (>90%) for DCM, which are all consistent with prior reports^12^. Of note, these symptoms are captured by the mJOA score, which we found to have high sensitivity (82%) and specificity (90%) with a cutoff of <= 16/18 for DCM; these results are similar to a Treanor et al. that found mJOA had 62% sensitivity and 90% specificity for DCM diagnosis at the same cutoff^14^. Our study also found that patient-reported UE weakness and arm pain were moderately associated with DCM, similar to results reported by Jiang et al.^12^ However, our finding that headaches are common in DCM (58%) differs greatly from the only available prior report (8%)^15^. PROMs demonstrated moderate discriminatory performance, including NDI, EQ-5D-5L, and EQ-VAS, which indicates that DCM substantially impairs activities of daily living and quality of life. However, patient-reported symptoms and questionnaires are highly subjective and can yield false positives in a range of clinical scenarios, including patients that over-report symptoms for various reasons (anxiety, drug-seeking, etc.) and those with orthopedic and neurological comorbidities, limiting their utility and highlighting the need for corroborating evidence.

Clinical signs and physical testing can arguably provide objective data to confirm the diagnosis of DCM, but in our data many of these tests tended to have modest diagnostic accuracy. Exceptions to this were self-paced velocity and our scored tandem gait assessment, which were highly sensitive (69% and 73% respectively) and moderately specific (79% and 57% respectively). Our tandem gait results performed similarly to several reports of similar assessments in other neurological conditions^16–20^. We created this tool^35^ as a modification of the 10-Step Tandem Gait Test, reported by Yoo et al. (2021), such that it can be performed in a clinical setting without specialized equipment^18^. Our scoring scheme increases the number of ordinal levels to 6 and requires simply counting the number of stumbles during the walk and observing for swaying. Self-selected walking velocity, which can be measured using a 6-, 10-, or 30-meter walk test^21^, slightly outperformed fast-paced walking velocity, similar to prior results^22^. However, this test is slightly more cumbersome and may be subjective, given the arbitrary nature of self-pacing. More generally, we found that certain physical tests had enhanced diagnostic performance when combined by summation, including UE reflexes (biceps, triceps, brachioradialis, inverted brachioradialis sign, and Tromner or Hoffman sign), manual motor testing of 5 key muscle groups (elbow flexion, finger extension, finger flexion, 1^st^ dorsal interosseous, and thumb opposition), and an abbreviated (9-item) version of the Berg Balance test. However, the Berg Balance test is lengthy, and we felt that tandem gait or standing on 1 foot both appeared to be more practical tests for routine practice. Reflex examination of the UEs showed good diagnostic performance, highlighting the importance of teaching this skill in medical education. Specifically, the Tromner sign, which is not widely utilized in North America, and the Hoffmann sign are similar exam maneuvers, that both demonstrate good sensitivity (47%, 48%, respectively)^23,24^. The Tromner sign is evoked by flicking the ventral surface of the middle finger’s terminal phalanx, while the Hoffmann sign is triggered by flicking the dorsal tip of the middle finger^23^. The most specific tests were Tromner and Babinski (98%), closely matching the results of a recent systematic review and meta-analysis, but Babinski has poor sensitivity^11^. Highly specific signs such as Babinski’s sign for DCM are valuable for confirming the diagnosis when present but if sensitivity is low, they carry little utility as a screening tool. In contrast, highly sensitive tests are better suited for initial screening but, if non-specific, require confirmatory testing to avoid false positives. All sensory modalities generally showed poor diagnostic performance in our data, which contradicts a recent report suggesting that proprioception is highly sensitive for DCM^25^. However, we found that testing of light touch in the UEs (C5-T1) has similar performance with pin prick and monofilaments, and slightly outperforms proprioception and vibration, suggesting that this simple test may be adequate for routine clinical assessments. However, it remains unclear how to best examine for subtle sensory dysfunction to corroborate this common subjective symptom, suggesting there may be a role for advanced neurophysiological techniques, such as contact heat evoked potentials (CHEPs), which appear to be highly sensitive to DCM^26,27^. Overall, our results indicate that certain physical tests, including reflex examination, manual muscle testing, and both gait and balance assessment, are useful for confirmation of DCM diagnosis, although these require moderate training and experience. Further research will be beneficial to determine if other physical tests can further enhance diagnostic accuracy.

In spite of being one of the most common central nervous system disorders, the diagnosis of DCM is frequently missed or delayed for a variety of reasons. There is a widespread lack of awareness of this condition among the general public and primary care practitioners, indicating a potential need for awareness campaigns and improved medical education. In addition, the clinical presentation of DCM is heterogeneous and existing evidence reports on a vast range of symptoms with conflicting results, failing to identify straightforward means to screen for this condition. Both primary care clinicians and specialists sometimes fail to perform or are not adequately trained to complete neurological assessments, potentially missing key findings. Historically, DCM was diagnosed by the presence of multiple symptoms, such as the triad of hand numbness, clumsiness, and walking imbalance, and obvious signs like spastic gait or wheelchair dependence^17^; however, these deficits are only present when DCM is at an advanced stage. Furthermore, the inability to diagnose milder cases of DCM led to very low estimates of prevalence, which in turn has perpetuated a lack of interest and awareness of this condition^9^. However, there is a growing recognition that mild DCM is common, while its onset is often insidious with intermittent symptoms and a lack of fulminant signs^2,6^. Primary care practitioners need to pay close attention to concerning symptoms, which should then prompt a thorough neurological exam and consideration of the need for imaging such as a cervical MRI and referral to an appropriate specialist, to confirm the diagnosis and direct treatment. Education of patients and primary care practitioners to increase symptom awareness could potentially be beneficial for earlier diagnosis, prompt treatment, and improved outcomes. However, educational interventions have shown limited benefit in other spine pathologies such as chronic low back pain^28^, suggesting that such educational programs requires thoughtful design and prospective study. Our findings help lay the groundwork for improving the early diagnosis of DCM by combining the most sensitive symptoms (e.g., neck pain, UE numbness/clumsiness/weakness, and gait imbalance) with a small set of simple neurological tests (e.g., reflexes, hand intrinsic strength, tandem gait), which constitute a pragmatic screening approach.

Efforts are underway to standardize the diagnosis of DCM^29^, but the lack of diagnostic criteria remains a major barrier to improving recognition and management of this condition^30^. Definitions of DCM in the literature vary widely, with many of the most important studies of this condition offering only vague inclusion criteria^31,32^. Some studies have been more specific, requiring patients to have one or more symptoms, one or more signs, and MRI evidence of spinal cord compression^7,24,33^, but there is even controversy on what imaging findings are required to confirm the diagnosis^4,34^. Furthermore, the presence of a confounding neurological condition has been occasionally specified as an exclusion criterion^7,35^. We agree with this general framework, and in fact used similar criteria in the current study as the “gold standard” to which each symptom and test was measured against. However, a lack of consensus currently exists on which symptoms and signs must be present to make a diagnosis. For example, it remains unclear if the presence of neck pain (without other symptoms) and one or more clinical signs (e.g. hyperreflexia), with concordant imaging findings, is sufficient to make the diagnosis of DCM. Our results contribute substantially to the evidence regarding diagnosis of DCM, providing sensitivity and specificity for a range of data, but validation is required to externally confirm results and reconcile differences with prior literature. Ultimately, we hope that this study contributes both to building consensus on how to optimally assess DCM and to the development of robust diagnostic criteria that can achieve consistent and earlier diagnosis.

Several limitations were present in this study that need to be recognized. First, our DCM patients and healthy subjects were approximately age-matched, but minor differences could lead to bias. Specifically, this could impact the diagnostic accuracy of symptoms and signs that are strongly affected by age (e.g. hand arthritis causing hand incoordination). We have completed a separate analysis of our healthy cohort that demonstrates several outcome measures vary with age^36^, but this had only minimal effects on sensitivity and specificity. The DCM cohort also demonstrated higher BMI, potentially indicating a lower level of general health and mobility, which could also bias results, but this also may be an effect of having DCM. Our recruitment method of inviting all DCM patients to participate reduced investigator-driven selection, but participation remained subject to logistical constraints, which may have biased the sample toward patients with mild-to-moderate disease as those with more severe disability often underwent a lengthy visit to discuss surgery and would not be willing to stay for a research study. Furthermore, participants were given the option to come back for another research visit, but those with upcoming surgery or severe mobility limitations were less likely to return. Nevertheless, our cohort included a balanced representation of mild, moderate, and severe DCM cases. The lack of blinding between imaging interpretation and clinical findings may also have introduced bias, as diagnosis of DCM in the context of subtle imaging findings is also controversial but we tried to standardize diagnosis through a final eligibility review by the first and senior authors^37^. We did not investigate certain other symptoms, such as Lhermitte’s sign, or physical tests, such as the 10-second grip and release test, due to time limitations. Furthermore, we purposefully included DCM and healthy subjects with minor but potentially confounding orthopedic and neurological conditions to reflect a real-world DCM population, but this could impact results if these conditions were over or under-represented compared to their general prevalence. However, we would argue that strict exclusion of minor neurological or orthopedic comorbidities lacks external validity, as this “messy” picture is typically what is found in the age group in which DCM is present. Furthermore, clinical studies with a “clean” control group that excludes any neurological symptom or diagnosis have a substantial risk of bias, since these conditions are equally likely in DCM patients but symptoms may be falsely attributed to DCM (e.g. hand numbness that is actually caused by carpal tunnel syndrome).

## Conclusions

**Figure 5:**
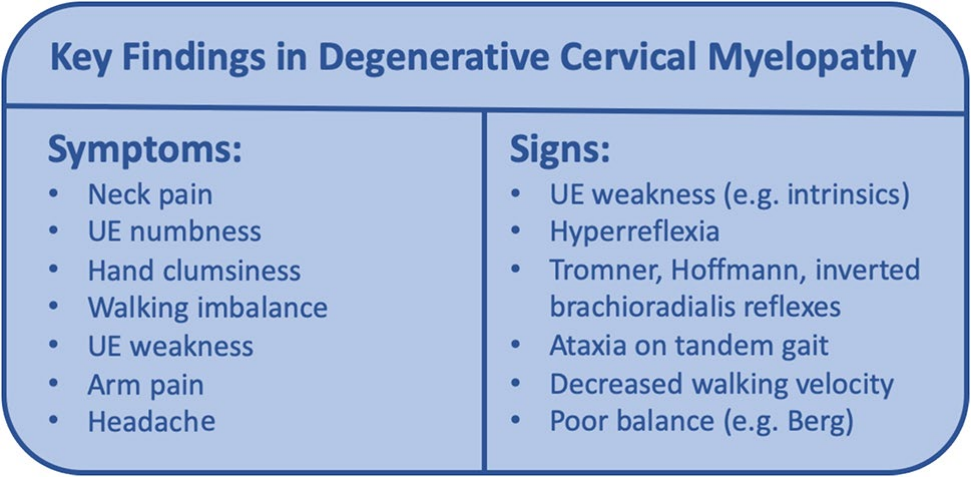
Key findings in degenerative cervical myelopathy.

This study evaluated a multitude of clinical symptoms and signs and identified a subset that accurately differentiates individuals with DCM from their healthy counterparts, in a large cohort of DCM and healthy subjects. Our data indicate that the group of symptoms—neck pain, UE numbness, hand clumsiness, walking imbalance, UE weakness, UE pain, and headache—best discriminate DCM from healthy controls (Figure 5). Among bedside tests, UE reflexes, UE manual motor testing, tandem gait, and walking velocity provided useful objective confirmation. Neck pain has high sensitivity but only moderate specificity, so should not be used alone; rather, its presence should prompt further questioning for other symptoms and consideration of a focused neurological exam to make a presumptive diagnosis, in which case cervical MRI and/or specialist referral should be performed. Our findings hold promise in contributing to earlier and more accurate diagnosis of DCM, which is imperative given worldwide challenges in effectively diagnosing and managing this condition. Future efforts should be directed toward the development and evaluation of screening tools, education of front-line clinicians, and the creation of detailed diagnostic criteria to achieve earlier diagnosis and standardized treatment of this important condition.

## Supplementary Information

Supplementary Information.

## Data Availability

The datasets generated during and/or analyzed during the current study are available from the corresponding author on reasonable request.
