# Supplementary material for: Assessing the diagnostic accuracy of symptoms and signs of degenerative cervical myelopathy: A prospective study
Source: Sci Rep. 2025 Oct 23;15:37043. doi: 10.1038/s41598-025-20928-4 (PMC12550042; doi:10.1038/s41598-025-20928-4)
Supplement: Supplementary file 1 — Supplementary Information. [file 41598_2025_20928_MOESM1_ESM.docx]

**Supplemental Tables:**

| **Symptom** | **Sensitivity** | **Specificity** | **Youden’s Index** | **P value** |
| --- | --- | --- | --- | --- |
| Neck Pain | 85% | 78% | 63% | <0.001 |
| Upper Extremity Numbness | 67% | 90% | 57% | <0.001 |
| Hand Incoordination | 58% | 92% | 50% | <0.001 |
| Gait Imbalance | 58% | 92% | 50% | <0.001 |
| Upper Extremity Weakness | 53% | 93% | 46% | <0.001 |
| Arm Pain | 51% | 90% | 41% | <0.001 |
| Back Pain | 72% | 67% | 40% | <0.001 |
| Headache | 58% | 77% | 36% | <0.001 |
| Lower Extremity Weakness | 39% | 95% | 34% | <0.001 |
| Urinary Dysfunction | 41% | 91% | 32% | 0.001 |
| Lower Extremity Numbness | 38% | 91% | 29% | <0.001 |
| Leg Pain | 40% | 84% | 24% | <0.001 |
| Fecal Incontinence | 12% | 98% | 10% | 0.01 |
| Sexual Dysfunction | 14% | 94% | 9% | 0.17 |
| Saddle Numbness | 6% | 98% | 4% | 0.12 |

**Supplementary Table 1. Diagnostic Accuracy of Symptoms in DCM.** Sensitivity, specificity, and Youden’s index values are presented as percentages. Statistical methods and error analysis adhered to standard diagnostic test evaluation guidelines. P values displayed were corrected for multiple comparisons using Benjamini-Hochberg procedure.

*Data were missing for headache (1 DCM), and sexual function (12 DCM and 12 HCS).*

| **Muscle Group / Function** | **Abbreviation** | **Function Assessed** |
| --- | --- | --- |
| **Upper Limb Muscles** |  |  |
| Shoulder Abduction | SAb | Lifting arm sideways with elbow bent |
| Elbow Flexion | EF | Bending at the elbow |
| Elbow Extension | EE | Straightening at the elbow |
| Wrist Flexion | WF | Bending the wrist downward |
| Wrist Extension | WE | Bending the wrist upward |
| Finger Flexion | FF | Bending fingers forward against resistance |
| Finger Extension | FE | Straightening fingers against resistance |
| Flexor Pollicis Longus | FPL | Bending the thumb at the proximal phalanges |
| Finger Abduction | FAb | Testing of of the 5^th^ digit (abductor digiti minimi) |
| Finger Adduction | FAd | Bringing fingers together |
| First Dorsal Interossei | 1DI | Moving index finger sideways |
| First to Fifth Digit Opposition | Opp | Strength of thumb to pinky with examiner attempting to separate |
| **Lower Limb Muscles** |  |  |
| Hip Flexion | HF | Lifting thigh forward |
| Hip Extension | HE | Bringing thigh backward |
| Hip Abduction | HAb | Bringing leg outward |
| Hip Adduction | HAd | Bringing leg inward |
| Knee Flexion | KF | Bending the knee back |
| Knee Extension | KE | Straightening the knee forward |
| Ankle Dorsiflexion | AD | Lifting foot upward |
| Ankle Eversion | AE | Turning foot outward |
| Ankle Inversion | AI | Turning foot inward |
| Ankle Plantarflexion | AP | Pointing foot downward, tested with patient standing |
| Extensor Hallucis Longus | EHL | Extending big toe |
| Flexor Hallucis Longus | FHL | Flexing or curling big toe down |
| **Grip Strength Assessments** |  |  |
| Grip Strength |  | Measured using Jamar dynamometers (average of 3 trials) |
| Pinch Strength |  | Measured between 1st and 2nd digits |
| Opposition Strength |  | Measured between 1st and 5th digits |
| Adduction Strength |  | Measured between 2nd and 3rd digits |

**Supplementary Table 2. Description of Muscle Groups Involved in Manual Motor Testing.**
